# The effect of honey in oral care intervention against chemotherapy-induced mucositis in pediatric cancer patients: a pilot study

**DOI:** 10.1186/s12906-024-04710-z

**Published:** 2024-12-18

**Authors:** Ikeu Nurhidayah, Yeni Rustina, Sutanto Priyo Hastono, Henny Suzana Mediani

**Affiliations:** 1 Department of Pediatric Nursing, Faculty of Nursing, Universitas Padjadjaran, Jalan Ir. Soekarno KM. 21, Jatinangor, Sumedang, West Java, 45363 Indonesia; 2 Department of Pediatric Nursing, Faculty of Nursing, Universitas Indonesia, Jalan Prof. Dr. Bahder Djohan, Depok, West Java 16424 Indonesia; 3 Department of Biostatistics and Population Studies, Faculty of Public Health, Universitas Indonesia, Jalan Prof. Dr. Bahder Djohan, Depok, West Java 16424 Indonesia

**Keywords:** Chemotherapy-induced mucositis, Children, Honey, Oral care

## Abstract

**Objective:**

Mucositis is one of the common side effects of chemotherapy. This study aimed to identify the effects of honey on oral care interventions to reduce mucositis scores among children undergoing chemotherapy.

**Methods:**

This pilot study was quasi-experimental with pre-post intervention with the control group. The study employed consecutive sampling, with 24 patients in the control and 24 in the intervention group. The intervention group received an oral care protocol using honey, whereas the control group received regular oral care. The data were collected using demographic information form and the Oral Assessment Guide (OAG) to assess mucositis. The intervention group received oral care intervention using honey (35 ml of honey applied topically in the oral cavity and 15 ml of honey used as a mouthwash and for lip care), which was given thrice daily for five days. The data were analyzed using percentage distributions, means, chi-square tests, dependent and independent t-tests, and multivariate analysis using analysis of covariance (ANCOVA).

**Results:**

The findings of this study showed that the intervention group experienced a significant reduction (-0.51 ± 0.66) in the average mucositis score after the intervention, whereas the control group experienced an increase in the mucositis score (3.84 ± 1.28) after controlling for confounding variables (*p* = 0.000).

**Conclusion:**

This study revealed that oral care with honey effectively reduced chemotherapy-induced mucositis in children with cancer. These findings suggest that oral care with honey should be used as a nursing intervention for chemotherapy patients.

**Trial registration:**

This clinical trial was retrospectively registered in the Australian New Zealand Clinical Trials Registry (ANZCTR) with registration number ACTRN12624001313527 (29/10/2024).

## Background

Cancer is a serious disease that endangers the health of children. Every year, the number of new cases of childhood cancer has increased significantly. According to the National Cancer Institutes, 15,590 children and adolescents will be diagnosed with cancer by 2021 [1]. According to World Health Organization (WHO) estimates, the incidence of cancer in children is approximately 4%, with 400,000 newly diagnosed children and an increase in cases of 110–130 per million children [2]. The majority of children with cancer (80%) were from developing or low or middle-income countries, with a total death rate of 90,000 cases [3–5]. However, children with cancer still have a low survival rate in developing countries, at less than 60% and even less than 30% [2, 6].

Childhood cancer treatment is intensive and continuous, intending to control the number and spread of cancer cells and increase survival rates [7]. The primary therapeutic modalities for treating childhood cancer are surgery, chemotherapy, and radiotherapy, with additional therapies including immunotherapy, photodynamic therapy, stem cell transplantation, and targeted therapy [8]. Chemotherapy is one of the most effective cancer treatments for children, particularly those with leukemia [9] and other types of cancer, including nasopharyngeal cancer, rhabdomyosarcoma, and lymphoma [7, 10, 11].

Chemotherapy involves administering cytotoxic drugs that destroy and inhibit the growth of cells that divide rapidly but cannot distinguish between cancer and normal cells [12]. Normal cells that divide rapidly, particularly mucosal cells in the oral-gastrointestinal tract, can experience chemotherapy side effects such as mucositis, nausea, and diarrhea [12, 13]. Harris conducted a systematic review and reported that side effects of chemotherapy in the mouth and gastrointestinal tract were the most common symptoms (69.6%) among the primary studies identified, indicating that these symptoms were most commonly reported by cancer patients receiving chemotherapy [14].

Oral mucositis is one of the most common side effects of chemotherapy [14, 15]. Mucositis is an inflammation and ulceration of the mucous membranes of the mouth. The prevalence of mucositis is 40–90% in children under 12 years old [11] and 75–80% in children who receive high-dose chemotherapy [16]. Mucositis symptoms range from mild to severe and may necessitate hospitalization. These symptoms include pain, erythema, edema, ulceration, bleeding, dry mouth, burning sensation, difficulty swallowing and speaking, and can impact all aspects of a child’s life [16].

Mucositis-related ulceration and pain can make eating and drinking difficult due to impaired chewing, swallowing, tasting food, and bad breath [17]. This can lead to decreased appetite and limited food and drink intake, putting children at risk of dehydration, weight loss, and nutritional changes [18]. Severe dietary changes can increase a child’s enteral and parenteral nutrition needs [19]. Significant dietary changes have several unintended consequences, including worsening clinical conditions and increasing the risk of infection. This exacerbates the child’s condition and can result in reduced chemotherapy doses, schedule delays, or even treatment discontinuation [20]. As a result, the effectiveness of chemotherapy becomes suboptimal, disrupting the remission period and reducing recovery and child survival [16, 19, 20]. Thereby increasing the cost and ultimately reducing the quality of life of children [21].

As healthcare professionals, nurses are responsible for providing high-quality nursing care for mucositis-induced chemotherapy. Numerous types of mucositis interventions are being researched and developed [22]. Friend’s systematic reviews suggest oral care is the best mucositis treatment [23]. However, some studies still debate the best oral care agent for preventing and treating mucositis.

Chlorhexidine is a widely and frequently used oral care agent. However, some studies advise against using this agent. Several studies have shown that 0.2% chlorhexidine is ineffective at preventing and treating mucositis [24]. Other agents, such as povidone-iodine, provide weak scientific evidence for preventing mucositis. Some studies suggest that povidone-iodine does not significantly reduce mucositis [25–29]. Previous studies have shown that compared to the cohorts using povidone-iodine gargle, the turmeric mouthwash group delayed and reduced the levels of radiation-induced oral mucositis, which was statistically significant at all time points [28]. Meanwhile, a meta-analysis study showed that of nine (9) oral care solutions for the prevention of oral mucositis, povidone-iodine was a less effective mouthwash agent for reducing chemotherapy-induced mucositis scores compared to other mouthwash agents such as curcumin, honey, and benzydamine [29].

Chlorhexidine and iodine are also less tolerated by children. Brown and Gupta noted that most patients experience an unpleasant taste when using these substances [30]. These substances should not be given over a long period. These products should not be consumed because they may alter the normal bacterial flora in the oral cavity and result in hyperthyroidism [30]. The agents chlorhexidine (0.12–0.2%) and Benzydamine (0.15%), according to the Multinational Association of Supportive Care in Cancer/International Society of Oral Oncology (MASCC/ISOO), have been linked to stinging or burning sensations [31]. Because of the stinging and burning sensation in the oral cavity of pediatric patients, randomized controlled trials concluded that patients require increased dilutions of chlorhexidine (to 33%) and Benzydamine (to 20%) [32]. Previous studies reported a severe stinging sensation in the mouth when benzydamine mouthwash (0.15%) was given, and 3 out of 4 patients dropped out of the study due to this side effect [32]. In addition, minor taste alterations were reported by pediatric patients when using chlorhexidine (0.2%) and benzydamine (0.15%) in 6% and 3–9% of patients, respectively [32].

Brown and Gupta suggested that studies should continue to look for agents for the treatment of mucositis that are less expensive, more effective, and have a pleasant taste when administered to children [33]. Some studies suggest that honey can treat mucositis [34, 35]. Honey is a product of the nectar of flowers that bees have aero-digested. Honey can be used to treat mucositis because honey contains the enzyme glucose oxidase, which converts glucose to glucose acid. These substances inhibit the growth of bacteria [22]. Honey also has a high osmolality, which leads to water extraction from bacterial cells, causing the bacteria to die. Honey also has low acidity (pH: 3.3 to 4.7), and bacteria find living in such conditions difficult [36].

The use of honey has a level of evidence in the clinical guidelines published by the MASCC/ISOO; honey is suggested to prevent mucositis in patients in the general population [31] and special pediatric populations [37]. The MASCC/ISOO panel also revealed that honey and photobiomodulation therapy has promising results in pediatric patients [37]. The MASCC/ISO, on the other hand, encourages further research into the effectiveness of honey against mucositis in the pediatric population.

Two studies were conducted to determine the effect of honey as a topical agent for reducing mucositis in adult patients receiving chemotherapy [34, 38]. According to a systematic review, honey or honey products can prevent chemotherapy-induced oral mucositis and are the best treatment for Grades I, II, and III patients. According to the findings of these studies, honey can help reduce mucositis in adults [35]. A systematic review study by Nurhidayah (2024) showed that three experimental studies identified honey’s effect in managing mucositis in the pediatric population [15, 22, 36, 39]. Most studies on using honey and its impact on managing mucositis were conducted in adults [40–44]. According to the MASCC/ISOO clinical recommendation, using agents such as honey in oral care interventions has a level of evidence suggested for preventing mucositis in general population patients and special pediatric populations [15, 37]. The MASCC/ISOO panel also believes that honey has promising potential in pediatric patients, but more study is needed to strengthen this evidence [37]. This study aimed to identify the effects of honey on oral care intervention to reduce mucositis scores among children undergoing chemotherapy. The study was carried out at the National Referral Hospital in Indonesia.

## Methods

### Design

This pilot study used a quasi-experiment pre and post-test with a control group. It was conducted at the Pediatric Haematology-Oncology Inpatient Unit in a national referral hospital in Indonesia between March 2011 and June 2013. This hospital is also a national reference for pediatric cancer management.

### Guiding theory

**Fig. 1:**
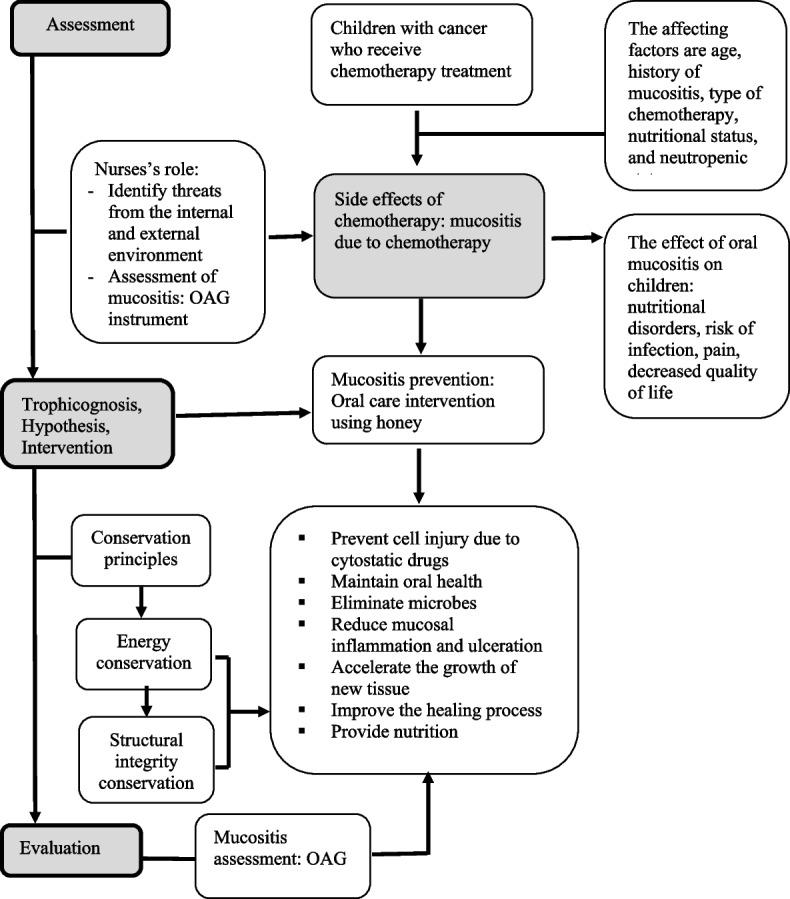
Levine’s conservation theory framework

This study was guided by Myra E. Levine’s conservation model theory (Fig. 1) [45–47]. Levine views children as open individuals who are always responsive to the environment. Children with cancer who are undergoing chemotherapy are viewed as individuals who adapt to internal and external threats. The presence of cancer cells that threaten normal cells is a threat from the internal environment, whereas side effects of chemotherapy and environmental exposure are threats from the outside. Chemotherapy-induced mucositis is one risk for children. The nurse suggests implementing a series of preventive procedures to avoid mucositis caused by chemotherapy.

Levine believes that nursing interventions must follow the principle of therapeutic intention. Therapeutic intention aims to facilitate the natural healing process of chemotherapy-induced mucositis, allow the body to autoregulate appropriately, and restore the child’s integrity and well-being. The honey-based oral care protocol is consistent with Levine’s therapeutic intention principle. As a result, including honey in the oral care protocol is intended to help children conserve energy while preserving structural integrity. Children can progress to personal and social integrity if energy conservation and structural integrity are achieved.

### Participants

**Fig. 2:**
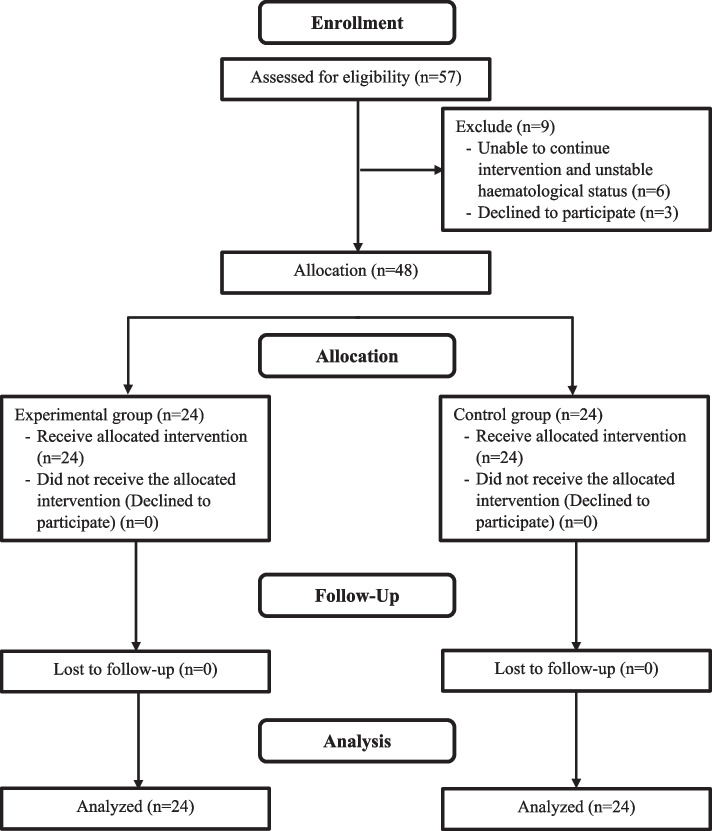
The flow diagram of study

Participants in this study were children with cancer who were undergoing chemotherapy at the children’s inpatient unit at one national referral hospital in Indonesia. Samples were taken by purposive sampling. Children who were receiving chemotherapy and who were at least two years old did not have impaired hepatic or renal functions. The patients had hematological values within the normal range and met the inclusion criteria. Children with stage 3–4 nasopharyngeal cancer who had difficulty opening their mouths and could not receive oral care interventions were excluded from this study. The estimated sample size can be determined by knowing previous research’s mean and standard deviation [48]. The sample size in this study was estimated with a 95% confidence interval and a statistical power of 80% using the sample formula for two independent means [49, 50]. According to the calculations, the minimum sample size for each group was 24 participants. A total of 57 respondents were included in this study, but nine respondents dropped out (six children were unable to continue the intervention due to unstable hematological status, and three children refused to continue the intervention because they did not like the taste of honey). Finally, 48 respondents were divided into an intervention group (24) and a control group (24). Figure 2 depicts the flow of this study.

### Intervention and procedures

This study included two groups of respondents: a control group and an intervention group. In the intervention groups, before implementing the intervention, nurses and doctors will educate parents and children for approximately 60 min about oral care with honey. In this session, parents will learn how to perform basic oral care (toothbrushing, mouthwashing, and lip care). The nurse will also teach about the benefits of honey and how to use it in oral care, such as how to apply honey to the mouth, make a honey mouth rinse, and use honey as a lip care agent in children’s oral care. The nurse demonstrated how to apply honey directly to the patient’s oral cavity and lips and make honey mouthwash. Nurses and doctors also advise parents to provide oral care to their children at least three (3) times per day using honey for five (5) days. The honey administration protocol used in this study was based on that used in the Montalebnejad study [44]. The intervention group received oral care through the use of honey thrice daily (30 min after breakfast, lunch, and before sleep) for five days. Each oral care session lasts between 15 and 20 min (including toothbrushing, mouthwashing, and lip care). For each oral care intervention, 35 mL of honey was applied topically in the oral cavity, and 15 mL was used as a mouthwash and lip care. The first application of honey in oral care to the patient was done by the nurse. The nurse will apply honey to the oral cavity, give the patient honey mouthwash, and smear honey on the lips. The next oral care application of honey was done by parents. The nurse provides parents with a daily activity book to monitor their compliance with the intervention. The book includes a daily oral care activity sheet, which must be completed every time parents provide oral care for their child. Apart from that, parental compliance is monitored by nurses directly observing parents’ actions in providing oral care intervention to children for five (5) days while the child is in the hospital.

In this study, Indonesian natural and standardized flower honey was administered to the intervention group, in addition to routine and standard care performed at the clinic after each chemotherapy session. The honey used was a flower honey from Indonesian forests that met Indonesian national standards. The honey contained 17–20% (maximum water content of 22%), 60% sugar reduction, 10% sucrose, 40 ml NaOH/kg acidity, and the diastase enzyme activity of 8 DNs (minimum 3 DNs). These Indonesian honey samples contained minerals (Ca, Na, P, Fe, Mg, and Mn), vitamins (B1, B2, B5, B6, and C), and enzymes [51].

The control group will receive routine oral care procedures. In the control group, nurses and doctors will provide a 60-minute educational session about oral care to parents and encourage parents to perform oral care on children routinely at least three (3) times a day (after breakfast, lunch, and dinner) for five (5) days using oral care agent prescribed by the doctor. In the control group, parents will routinely perform oral care using mouthwash and medicine prescribed by the doctor for each patient following hospital guidelines. In this study, the control group used 0.9% NaCl, chlorhexidine, and benzydamine as their oral care agent. The nurse will observe and monitor the implementation of oral care in the control group. The nurse also provides parents in the control group with a daily oral care activity sheet, which must be completed every time parents provide oral care for their children. Nurse continue to encourage parents and patients in the control group to adhere to oral care during treatment after chemotherapy. Aside from that, parental compliance is monitored by nurses who directly observe parents’ actions when providing oral care intervention to children for five (5) days while the child is in the hospital.

### Instrument

In this study, the children’s characteristics included age, sex, previous history of mucositis, type of cancer, type of chemotherapy, nutritional status, and neutropenic status. To obtain nutritional status data, anthropometric measurements were taken by measuring the child’s height and weight and comparing the results to the World Health Organization(WHO)’s standard body mass index (BMI) per age [52]. Documentation studies were also conducted to examine the findings of laboratory tests on liver and renal function and complete peripheral blood counts, leukocyte counts, and neutropenia status. Mucositis was evaluated using the Oral Assessment Guide (OAG) questionnaire developed by Eilers [53, 54]. The Oral Assessment Guide (OAG) consists of eight parameters: mucous membranes, lips, tongue, gingiva, and teeth; functional and subjective assessments of voice, salivary gland function, and swallowing ability. The assessment is described on a scale of 1–3 for each parameter. The evaluation was performed through observation, visual examination, palpation, and auditory examination. The overall score is calculated by adding the values of each assessment parameter. The lowest score for mucositis was 8, and the highest was 24.

### Assessment

Mucositis was measured twice. Mucositis was measured by three nurses and one doctor who had received training and had equalized perceptions. In this study, an inter-observer reliability test was conducted to achieve consensus between data collectors. The consensus aims to equalize perceptions and assumptions between data collectors so that all data collectors have the same interpretation of the parameters to be observed. In this study, the inter-observer reliability test was carried out using a type of test for a numerical scale. Testing inter-observer reliability for numerical data can use Pearson’s coefficient correlation for a judge inter-reliability test. Suppose the p-value is less than alpha (α) and the reliability coefficient (r) is more than 0.80, it is considered that there is a strong/perfect agreement between the researcher and the numerator significantly [54]. The inter-observer reliability test in this study was conducted on five data viewers and obtained a p-value of 0.001 and a Pearson correlation coefficient (r) of 0.878. Based on these results, it can be concluded that there is a significant agreement (similarity of perception) regarding the observed mucositis score between the researcher and the research assistant (numerator), with a very strong/perfect level of agreement.

The initial mucositis score (baseline/pretest) in the intervention group was measured before respondents received chemotherapy drugs. Then, the measurement of the second mucositis score in the intervention group was carried out on the sixth day of hospitalization or one day after the child had completed the five (5) days intervention program. In the control group, the first mucositis score data collection (baseline) was taken before the respondent received chemotherapy drugs. Then, the second mucositis score data collection (post-test) was taken on the sixth day of hospitalization.

### Data analysis

#### Univariate and bivariate analyses

Univariate analysis was used to describe the respondents’ characteristics based on their previous mucositis experience, type of chemotherapy agent, nutritional status, type of cancer, and mucositis scores before and after chemotherapy. The homogeneity test using chi-square was used to determine the equality between the control and intervention groups on the variables of age, gender, history of mucositis, type of chemotherapy agent, nutritional status, and type of cancer. The bivariate analysis used a dependent and independent t-test.

#### Multivariate

In this study, multivariate analysis using the analysis of covariance (ANCOVA) test was used to determine whether the effect of oral care with honey on reducing the mucositis score was influenced by confounding variables (covariance). The ANCOVA test aims to improve the precision of group comparisons by calculating the variation in important prognostic variables or determining whether there is a relationship between covariates and response variables and the effect of treatment differences on response variables [55]. Previous experience with mucositis, nutritional status, type of chemotherapy, and type of malignancy were considered confounding variables in this study. The dependent variable in this study was the post-intervention mucositis score and the difference in mucositis score, and the independent variable was the honey administration intervention in oral care. The ANCOVA test results allow interpretation of the main effect after controlling for covariate effects.

#### Ethical consideration

This study was approved by the Institutional Ethics Committee of the Faculty of Nursing, University of Indonesia, and official permission was obtained from Dr. Cipto Mangunkusumo National Hospital (letter number: 315/UKA/I/IV/2011). The study procedures followed the guidelines of the Helsinki Declaration. Participation in the survey was contingent voluntarily, and as a result, no personal data of the patients were collected, thus preserving their confidentiality and privacy. After informing the children and their families of the purpose of the research and the objectives for which the data would be used, each participant provided verbal and written informed consent. Their parents’ written informed consent was obtained for children under the age of seven. Both children and their parents’ written informed consent was obtained for children aged 7–18 years old. The participants’ anonymity and confidentiality were guaranteed.

## Results

### Respondents’ characteristics

Table 1 shows the characteristics of the respondents.

**Table 1:** Characteristics of the respondents based on gender, previous mucositis experience, nutritional status, type of chemotherapy, and type of malignancy

Variables	Control Group (*n* = 24)		Intervention group (*n* = 24)		*p*-value
	F	%	f	%	
**Gender**					
Male	15	62.5	15	62.5	*p* = 1.000
Female	9	37.5	9	37.5	
**Previous mucositis experienced**:					
Yes	17	70.80	19	79.20	*p* = 0.793
No	7	29.20	5	20.80	
**Nutritional Status**:					
Normal	14	58.30	14	58.30	*p* = 1.000
Malnutrition	10	41.70	10	41.70	
**Type of Chemotherapy**:					
Mild-moderate Mucosatoxic	4	16.70	5	20.80	*p* = 1.000
High mucosatoxic	20	83.30	19	79.20	
**Type of malignancies**:					
Solid cancer	10	41.57	7	29.17	*p* = 0.564
Hematological cancer	14	58.33	17	70.83	

Table 1 shows that the two groups are similar. Most of the respondents in both groups were male (62.5%) and had previously experienced mucositis (79.2%) in the intervention group and 70.80% in the control group). The nutritional status of the two groups was also comparable, with the majority of respondents in both groups having a normal nutritional status (58.30%). Almost all respondents in the control (83.30%) and intervention (79.20%) groups had received high mucosatoxic chemotherapy. Most respondents in both groups had hematological cancer (leukemia and lymphoma). The study revealed no significant differences between the intervention and control groups in terms of previous mucositis experience (*p* = 0.793), nutritional status (*p* = 1.000), mucosatoxic potential (*p* = 1.000), or cancer type (*p* = 0.564).

**Table 2:** Characteristics of the respondents based on age

Variable	Group	Mean	SD	*N*	Minimal-Maximal	95% CI	*p*-value
Age (year)	Intervention	8.13	0.92	24	3–16	6.20–10.05	0.873
	Control	7.92	0.89	24	3–16	6.06–9.78	

Table 2 shows that the control and intervention groups were equivalent in age. The analysis showed that the mean age of the intervention group was 8.13 ± 0.92 years, and the control group was 7.92 ± 0.89 years. There was no significant difference in the age of respondents in both groups (*p* = 0.873).

### Mucositis score before intervention (baseline)

**Table 3:** Mucositis scores before intervention in the two groups

Variable	Group	Mean	SD	*N*	Minimal-Maximal	95% CI	*p*-value
Mucositis Score	Intervention	8.83	0.64	24	8–10	8.56–9.10	0.657
	Control	8.92	0.65	24	8–10	8.64–9.19	

Table 3 shows that the average mucositis score before intervention in the intervention group was 8.83 ± 0.64, while it was 8.92 ± 0.64 in the control group (*p* = 0.657). These findings indicate no statistically significant difference in baseline mucositis scores between the control and intervention groups.

### Comparison of mucositis scores before and after intervention in both groups

**Table 4:** Comparison of mucositis scores before and after the intervention in both groups

Variable	Group	Measurement	Mean	Standard Deviation	*p*-value
Mucositis score	Intervention	Before	8.83	0.64	0.002*
		After	8.38	0.49	
	Control	Before	8.92	0.65	0.000*
		After	12.71	1.43	

*) is significant at α < 0.05

Table 4 shows a significant decrease in mucositis scores after intervention in the intervention group (8.83 ± 0.64 to 8.38 ± 0.49) (*p* = 0.002). Moreover, there was a significant increase in mucositis scores in the control group at the second measurement (12.71 ± 1.43) compared with the baseline score (8.92 ± 0.65) (*p* = 0.000).

### Comparison of mucositis scores after the intervention in the two groups

**Table 5:** Comparison of mucositis scores after the intervention in the two groups

Variables	Group	Mean	Standard Deviation	*p*-value
Mucositis score after intervention	Intervention	8.38	0,49	0.000*
	Control	12.71	1,43	

*) is significant at α < 0.05

Table 5 shows a significant difference in mucositis scores between the two groups after intervention (*p* = 0.000). The mean mucositis score was 8.38 ± 0.49 in the intervention group and 12.71 ± 1.43 in the control group.

### The mean difference in mucositis between the intervention and control groups after intervention

**Table 6:** The mean difference in the mucositis score between the intervention and control groups

Variables	Group	Mean	Standard Deviation	*p*-value
Mean Differences	Intervention	−0.46	0.66	0.000*
	Control	3.79	1.28	

*) is significant at α < 0.05

Table 6 shows that the intervention group had a −0.46 mean difference with a standard deviation of 0.66. The minus (-) value in this result indicates that mucositis scores in the intervention group decreased in the second measurement. Moreover, the mean difference in mucositis scores in the control group was 3.79, with a standard deviation of 1.28. This positive (+) value indicates that in the control group, the mucositis score increased in the second measurement. Further analysis revealed a significant difference in the mean difference in mucositis scores between the intervention and control groups. Based on these findings, it was concluded that, with a 95% confidence level, oral care interventions using honey effectively reduce chemotherapy-induced mucositis, with an average reduction of 0.46.

### Multivariate analysis of potential confounding variables for the effect of the intervention on reducing mucositis score based on the ANCOVA

**Table 7:** Multivariate analysis of potential confounding variables against the intervention effect on mucositis scores based on ANCOVA

Parameters	B	*p*-value
*Intercept*	−0.92	0.087
Previous mucositis experienced	0.31	0.380
Type of Chemotherapy Agent	−0.17	0.658
Nutritional Status	−0.04	0.904
Type of malignancies	0.53	0.100
Oral care with honey intervention**	4.35	0.000*

*) α = 0,05; **) Partial Eta Square = 0.84

Table 7 shows that, based on the ANCOVA results, the p-value for the honey intervention was 0.000. After the intervention, oral care intervention with honey significantly impacted the mucositis score, even after we controlled for four confounding variables: previous mucositis experience, type of chemotherapy, nutritional status, and type of cancer. As a result, at the 95% confidence level, it can be concluded that the honey intervention in oral care significantly affects the mucositis score after the intervention. In the table above, the partial eta squared value for the honey intervention variable is 0.84. This value represents the magnitude of the effect of the honey intervention on mucositis scores after the intervention, which was 84%. These findings indicate that, at a 95% confidence level, administering honey in oral care reduces chemotherapy-induced mucositis by 84%.

**Table 8:** Multivariate analysis of the difference in mucositis severity score after controlling for confounding variables

Variable	Group	Before Control by Confounding Variables		After Control by Confounding Variables	
		Mean	Standard Deviation	Adjusting Mean	Standard Deviation
Mucositis Score	Intervention	8.38	0.49	8.37	0.23
	Control	12.71	1.43	12.72	0.23

Table 8 shows that the average mucositis score in the intervention group before controlling for confounding variables was 8.38, with a standard deviation of 0.49. After controlling for confounding variables, the adjusted mean score averaged 8.37 with a standard deviation of 0.23. This indicates a shift in the average value of 0.01. The average change value was minimal, indicating that the confounding variable had a minor and insignificant effect on the mucositis score following the intervention. Similarly, the control group had an average mucositis score of 12.71 with a standard deviation of 1.43 before controlling for confounding variables, and it became 12.72 with a standard deviation of 0.23 after controlling for confounding variables (adjusting the mean). The change in the average mucositis score in the control group was also minimal (0.01), implying that the confounding variable had only a minor and insignificant influence on the mucositis score in both groups.

**Table 9:** Multivariate analysis of the difference in mucositis score after controlling for confounding variables

Variable	Groups	Before Control by Confounding Variables		After Control by Confounding Variables	
		Mean	Standard Deviation	Adjusting Mean	Standard Deviation
Mean Difference	Intervention	−0.46	0.66	−0.51	0.21
	Control	3.79	1.28	3.84	0.21

Table 9 shows that the mean difference (decrease) in mucositis scores in the intervention group before controlling for confounding variables was 0.46. After controlling for confounding variables, the score decreased to 0.51. The adjusted mean value showed a more significant decrease than the value before controlling for confounding variables. Moreover, in the control group, the mean difference in mucositis scores before controlling for confounding variables was 3.79; after controlling for confounding variables, it was 3.84. A positive value (+) indicates an increase in mucositis score in the control group. After controlling for confounding variables, there was an increase in the difference in mucositis scores in the control group from 3.79 to 3.84. Overall, confounding variables did not significantly influence the mucositis scores in either group.

## Discussion

### Principal findings

This study determined the effects of honey in oral care interventions on chemotherapy-induced mucositis in children with cancer who were receiving chemotherapy. The statistical analysis revealed that the intervention group had a more significant decrease in mucositis scores than the control group. This study concluded that using honey as an oral care intervention effectively reduced chemotherapy-induced mucositis. These findings are consistent with previous studies that concluded that, in the honey treatment group, there was a significant reduction in oral mucositis associated with Candida and aerobic pathogenic bacterial infections [36]. The severity of oral mucositis in children in the honey group was significantly lower than that in the control group. The honey group recovered from mucositis significantly faster than the control group [22]. Another study concluded that honey significantly reduced the grade of chemoradiotherapy-induced mucositis in patients with head and neck cancer [40].

The study revealed no significant differences between the intervention and control groups regarding age, previous mucositis experience, nutritional status, mucosatoxic potential, or cancer type. Previous studies showed no significant differences in these variables regarding the effect of honey in reducing oral mucositis [36, 56–58]. In this study, oral care intervention with honey significantly impacted the mucositis scores after the intervention, even after controlling for four confounding variables, including previous experience with mucositis, type of chemotherapy, nutritional status, and cancer type. Honey is multifunctional and can significantly reduce mucositis, unlike other mouthwash agents (chlorhexidine, povidone iodine, or Benzydamine HCL). Several studies have shown that the administration of chlorhexidine, povidone iodine, and Benzydamine HCL is ineffective at reducing mucositis caused by chemotherapy. In addition, these agents should not be used indefinitely because they disrupt the normal flora of the mouth and cause irritation [59–63]. Honey has multiple functions because it has antioxidant, anti-inflammatory, antimicrobial, antifungal, and debridement effects; it stimulates tissue growth and repair and stimulates the body’s immune response so that wounds can recover more quickly [39, 57, 64, 65].

Providing honey to patients undergoing chemotherapy will help at every stage of mucositis pathophysiology. Chemotherapeutic agents can damage deoxyribonucleic acid (DNA) and cause the formation of reactive oxygen species (ROS) during the first phase of mucositis [21]. ROS causes tissue damage and initiates the inflammatory process. Giving honey immediately after chemotherapy may help to reduce ROS activation [66, 67]. This is due to the presence of antioxidants in honey. Honey contains antioxidant substances such as glucose oxidase, catalase, ascorbic acid (vitamin C), flavonoids, phenols, carotenoid derivatives, amino acids, and melanoidins [68]. These substances protect cells from oxidative stress caused by free radical production. As a result, providing honey immediately after chemotherapy is expected to prevent mucosal cell damage. Furthermore, honey inhibits proinflammatory activation agents in mucositis’s second and third phases [69, 70].

Compared to previous studies, this study’s findings suggest that using honey in oral care effectively reduces mucositis scores in children undergoing chemotherapy. A study investigating the effect of chlorhexidine in oral care to reduce mucositis scores discovered that chlorhexidine could increase mucositis scores [71, 72]. Another study on the impact of povidone-iodine on oral mucositis management in chemotherapy patients found that povidone-iodine was ineffective for oral mucositis management in children [25]. Another study shows that chlorhexidine in adults increased the mucositis score by 0.50, whereas using a magic solution (lidocaine solution, diphenhydramine hydrochloride, and aluminum hydrochloride) only reduced the mucositis score by −0.10 [71, 73]. Therefore, in the current study, the ability of honey to effectively reduce the mucositis score was − 0.51, considering that several previous studies using oral care agents other than honey showed smaller reductions than the present study.

Using honey as an oral care intervention during and after chemotherapy reduces mucositis by accelerating healing [35]. This is due to hydrogen peroxide in honey, which activates proteases. Protease activation causes debridement, increases subcutaneous blood flow in ischemic tissue, stimulates new tissue growth, and strengthens the anti-inflammatory response. Furthermore, honey accelerates new tissue formation by increasing fibroblast formation [22, 36, 41, 74]. The nutrients and energy in honey also provide nutrients to vital organs, epithelial cells, and macrophages. Moreover, the body’s healing ability increases by forming new mucosal epithelial cells [22, 36, 41, 74]. The current study’s findings provide information regarding honey’s effectiveness in oral care interventions against chemotherapy-induced mucositis in pediatric cancer patients.

### Implications of the study

Mucositis is a common side effect of chemotherapy in children. Chemotherapy-induced mucositis can cause physiological, functional, and social disturbances, decreasing the quality of life of children with cancer. The findings of this study indicate that oral health care using appropriate agents is a simple, inexpensive, and effective intervention. To date, oral care for children undergoing chemotherapy has received little attention from nurses and families. Indeed, various research findings indicate that oral care is an essential intervention for reducing mucositis in children.

The natural and straightforward agents used in oral care still need to be better socialized by nurses and parents. So far, artificial mouthwash agents such as iodine, chlorhexidine, or Benzydamine have been used as oral care agents for children receiving chemotherapy, even though various studies do not recommend using these agents for long periods due to the risk of irritation and disruption of the normal flora of the oral cavity. They are difficult to administer to children. Children and young children often refuse because of stings if lesions are present in their mouths; these patients do not taste well and should not be swallowed. Several studies, including this study, have evaluated using the natural agent honey, which effectively reduces mucositis during chemotherapy. Honey is also a well-known and easily accessible substance among families. Most cancer families know that honey has health benefits for their children, so they are familiar with it. In addition, honey has a complete nutritional content, tastes good, is sweet, and can be swallowed by children, making it easier to give to children than other oral care agents.

Children with poor oral health may have difficulty eating, drinking, chewing, swallowing, pain, or speaking. If the child is left unchecked, this will undoubtedly reduce the child’s quality of life. Hence, nurses, particularly pediatric specialist nurses, are responsible for providing quality nursing care by implementing oral care protocols using appropriate agents such as honey regularly and disciplined in the hope that mucositis caused by chemotherapy can be minimized. The findings of this study can be applied as an innovation in nursing care to prevent mucositis in children. Pediatric nurses are responsible for educating parents about the importance of regular oral care for children to prevent mucositis caused by chemotherapy. According to the principles of enabling and empowering family-centered care, pediatric nurses can empower parents to maintain their children’s oral health before, during, and after chemotherapy. Nurses need to empower parents to facilitate oral care in children with cancer through the use of agents, such as honey, that are scientifically proven to reduce mucositis.

## Conclusion

A honey-based oral care intervention can be used to prevent chemotherapy-induced mucositis in children with cancer who are receiving chemotherapy. The study included 48 children divided into two groups: intervention and control. This study concluded that after the intervention, there was a decrease in mucositis scores in the honey intervention group. At the same time, there was an increase in mucositis scores in the control group. The study’s findings suggest that oral care using honey as a nursing intervention significantly reduces chemotherapy-induced mucositis in the pediatric cancer patient population.

### Limitations

This study was conducted on cancer patients in general, with no distinction made based on the type of child cancer or a specific age group, so the outcome of the intervention may differ if it is conducted on particular types of cancer or specific age groups of children. Apart from that, the control group in this study received standard oral care in the hospital using oral care agents prescribed by the doctor, including NaCl 0.9%, benzydamine, and chlorhexidine, so the oral care agents in the control group were not specific to using one particular oral care agent. This is one of the study’s limitations, which is intended to be addressed in future studies by comparing honey to a specific oral care agent. However, this study is very important and useful for contributing to knowledge and evidence-based practice in the management of chemotherapy-induced mucositis in children with cancer.

## Data Availability

All data generated or analyzed during this study are included in this published article. The datasets generated and/or analyzed during the current study are available in the Universitas Indonesia repository or from the corresponding author at reasonable request.
